# Cold-Induced Urticarias with Familial Background: Clinical Spectrum, Pathogenesis, and Diagnostic Challenges

**DOI:** 10.3390/diagnostics15243195

**Published:** 2025-12-14

**Authors:** Nan Zhou, Yuxiang Zhi

**Affiliations:** Department of Allergy & Clinical Immunology, National Clinical Research Center for Immunologic Diseases, Peking Union Medical College Hospital, Chinese Academy of Medical Sciences & Peking Union Medical College, Beijing 100730, China

**Keywords:** familial cold urticaria, familial atypical cold urticaria (FACU), familial cold autoinflammatory syndromes (FCAS), NLRP3, NLRP12, NLRC4, PLCG2, F12, FACAS, acquired cold urticaria (ACU)

## Abstract

**Background:** Familial cold urticarias (FCU) are a group of rare hereditary disorders triggered by exposure to low temperatures. Their pathogenesis is complex, involving mast cell activation, inflammasome dysregulation, and abnormalities of the kallikrein–kinin system. This review aims to summarize the genetic classification, molecular mechanisms, and clinical implications of FCU in diagnosis and management. **Methods:** Recent literature was reviewed to outline the clinical and molecular characteristics of familial atypical cold urticaria (FACU), familial cold autoinflammatory syndromes (FCAS; including NLRP3-, NLRP12-, NLRC4-, and PLCG2-related subtypes), FXII-associated cold autoinflammatory syndrome (FACAS), and familial predisposed acquired cold urticaria (FP-ACU). Mechanistic clues and diagnostic strategies were analyzed, emphasizing the integration of clinical features with molecular findings. **Results:** Distinct FCU subtypes exhibit defined genetic bases: gain-of-function mutations in *NLRP3*, *NLRP12*, and *NLRC4* result in inflammasome hyperactivation; in-frame deletions in *PLCG2* lead to temperature-dependent immune signaling dysregulation; and heterozygous *F12* variants link contact activation with inflammatory cascades. Combining cold stimulation tests, inflammatory biomarkers, and targeted genetic sequencing enables precise molecular stratification. **Conclusions:** Molecular subclassification of FCU improves diagnostic accuracy and informs targeted therapy. Future research should focus on the interplay between cold-sensing ion channels, mast cell activation, and inflammasome signaling to advance precision diagnosis and individualized treatment of cold-induced urticarias.

## 1. Introduction

Cold urticaria (ColdU) is a subtype of chronic inducible urticaria characterized by the development of wheals, erythema, or angioedema upon exposure to cold air, cold water, or low-temperature surfaces. Severe cases may present with laryngeal edema or systemic reactions. Etiologically, ColdU is classified into two major forms: acquired cold urticaria (ACU) and cold urticaria with hereditary or familial background. For clarity within this review, we use the term ‘familial cold urticaria (FCU)’ as a descriptive umbrella for these hereditary forms [1,2]. In allergy and clinical immunology practice, ACU typically represents an IgE- or mast-cell-mediated reaction that responds well to second-generation H1-antihistamines. However, a subset of patients presenting with cold-induced wheals are now recognized to have genetic or familial forms of the disease, in which the underlying mechanisms involve innate immune dysregulation rather than IgE-mediated pathways—particularly the inflammasome, receptor signaling, and contact activation systems [3,4,5]. In summary, these differences distinguish ACU from the broader, genetically driven spectrum of FCU.

With advances in molecular immunology and genetics, the spectrum of hereditary and familial ColdU has become increasingly well defined. Familial atypical cold urticaria (FACU), first described in 2009, is characterized by early onset, lifelong persistence, hypersensitivity to atmospheric cold with a negative cold stimulation test (CSTT), and the absence of systemic inflammation [6]; familial cold autoinflammatory syndrome (FCAS), classically caused by gain-of-function mutations in *NLRP3* with activation of the NLRP3 inflammasome, presents with cold-induced urticarial rash, fever, arthralgia, and elevated inflammatory markers [3]. Subsequent reports have identified analogous cold-triggered phenotypes driven by mutations in *NLRP12* and *NLRC4*, which activate the NLRP12 and NLRC4 inflammasomes, respectively, thereby broadening the FCAS disease spectrum [7,8]. In contrast, PLCG2-associated antibody deficiency and immune dysregulation (PLAID) manifests with cold-induced urticarial lesions and humoral immunodeficiency, suggesting that adaptive immune signaling may also participate in cold reactivity [9]. More recently, FXII-associated cold autoinflammatory syndrome (FACAS), linked to *F12* variants, has been proposed as a distinct entity connecting cold-induced inflammation to the contact activation–bradykinin pathway regulating vascular permeability [10].

Beyond monogenic disorders, a group of patients with a familial tendency toward mast-cell mediated cold reactivity has been recognized. These individuals typically exhibit a positive CSTT, favorable response to antihistamines, and disease fluctuations with seasonal or emotional stress. Although their presentation mirrors typical ACU, the clear familial pattern contrasts with the way ACU is generally regarded as a sporadic condition. Their genetic background likely involves polygenic susceptibility that lowers the activation threshold of mast cells, leading to cold-triggered urticarial reactions [2,5,11]. To distinguish this form from classical autoinflammatory subtypes, we introduce the working term familial predisposed acquired cold urticaria (FP-ACU).

This review aims to comprehensively summarize FCU entities—including FACU, FCAS, PLAID, FACAS, and FP-ACU—highlighting their clinical heterogeneity, molecular mechanisms, and diagnostic challenges, and to delineate the boundary between clinically “allergic” (IgE-mediated) and autoinflammatory forms of ColdU.

Recent advances in molecular immunology have revealed that FCU represents a mechanistically heterogeneous spectrum. Its pathogenesis can be broadly categorized into four major immune axes: (1) reduced mast-cell activation threshold and thermo-sensory dysfunction; (2) inflammasome-driven autoinflammation; (3) temperature-dependent dysregulation of immune signaling pathways such as PLCG2; and (4) contact activation–bradykinin system abnormalities. Aberrations in these axes ultimately converge on mast-cell degranulation, inflammatory mediator release, and increased vascular permeability, leading to the characteristic urticarial response upon cold exposure. Table 1 provides an overview of the classification, molecular basis, and management of familial cold urticarias (FCU), serving as a practical reference for differential diagnosis.

## 2. Mast Cell Activation and Thermo-Sensory Dysregulation in FCU

### 2.1. FACU

FACU was first described by Gandhi et al. in 2009 [6]. Patients typically develop symptoms during childhood or adolescence, presenting with wheals, erythema, and localized swelling shortly after exposure to cold air, but without fever or systemic inflammation. The CSTT is usually negative. In a few cases, syncope has been reported after withdrawal from cold water. Most patients develop cutaneous symptoms within five minutes of exposure, which distinguishes FACU from delayed familial cold urticaria, where wheals typically appear 9–18 h after exposure [3,12]. The duration of symptoms ranges from 12 min to 24 h, depending on exposure intensity and treatment strategy, suggesting an individualized temperature threshold. Reported cases remain limited, mostly from European and North American populations. Epidemiologic data from ColdU cohorts suggest a higher prevalence in colder climates, though this has not been systematically quantified.

The molecular basis of FACU remains unclear, but patients respond well to second-generation H1 antihistamines. Skin biopsies show dermal mast cell infiltration and degranulation after cold challenge, supporting a mast-cell-mediated mechanism [6].

Diagnosis relies primarily on clinical history and findings—urticarial or angioedematous eruptions following cold-air or low-temperature exposure, in combination with a negative CSTT. The CSTT can be performed using an ice cube (0–4 °C) or TempTest device to differentiate from ACU [13]. The evaporative cooling test may further clarify the reaction pattern: applying room-temperature water under occlusion prevents evaporation and wheal formation, whereas exposing the site to compressed air induces localized erythema and wheals, indicating an evaporative cooling-dependent mechanism [6].

### 2.2. FP-ACU

In clinical practice, a subset of patients with a familial atopic background present with typical cold-induced wheals, a positive CSTT, and a good response to second-generation antihistamines, but without identifiable monogenic mutations. These cases, designated as FP-ACU, show overlapping clinical and immunologic features between ACU and FCU. Some studies have suggested that cold-induced urticaria may be associated with dysregulation of the transient receptor potential subtype M8 (TRPM8) [11].

The genetic susceptibility underlying FP-ACU is likely polygenic, resembling that of CSU. Candidate loci include immune-regulatory and cytokine-signaling polymorphisms in IFN-γ, IL-6, IL-17RA, IL-10, TGF-β, TNF, PTPN22, IL-1, IL-2, and HLA class I and II alleles [5,14].

## 3. Inflammasome-Related FCAS and PLCG2-Associated Cold-Induced Immuno-Dysregulation

Inflammasome-related FCAS constitute the mildest form within the cryopyrin-associated periodic syndrome (CAPS) spectrum. These rare systemic autoinflammatory diseases result from aberrant inflammasome activation and are mainly reported in European and North American populations [15]. Pathogenic variants in NLRP3, NLRP12, PLCG2, and NLRC4 underlie distinct subtypes, all characterized by cold-induced cutaneous inflammation and variable systemic involvement.

### 3.1. NLRP3-FCAS1

Gain-of-function mutations in the *NLRP3* gene cause the CAPS spectrum, which includes FCAS (OMIM 120100), Muckle–Wells syndrome (MWS, OMIM 191900), and neonatal-onset multisystem inflammatory disease (NOMID, OMIM 607115). Among these, FCAS represents the mildest clinical phenotype, historically referred to as ‘familial cold urticaria’ [16,17].

FCAS typically presents in infancy, with recurrent episodes of rash, fever, and arthralgia occurring 1–2 h after exposure to cold. Attacks may also involve chills, headache, myalgia, and conjunctivitis, usually resolving within 24 h and rarely life-threatening [18].

At the molecular level, activation of the NLRP3 inflammasome recruits ASC (apoptosis-associated speck-like protein), leading to Caspase-1 activation, Gasdermin D cleavage, and pyroptosis, accompanied by the maturation and secretion of IL-1β and IL-18. These inflammatory mediators trigger systemic inflammation characterized by cold-induced urticarial rash, fever, and joint pain, with elevated CRP and SAA, normal IgE, and negative CSTT [16,19].

Several adult-onset cases have been linked to low-level somatic NLRP3 mosaicism [20,21]. Because key immune cells in the circulation can activate the inflammasome, even a minute proportion of mutant leukocytes may exert pathogenic effects. Therefore, when routine sequencing is negative, high-depth next-generation sequencing (NGS) is essential for detecting low-frequency mosaic variants and achieving accurate molecular diagnosis [22,23].

### 3.2. NLRP12 FCAS2

FCAS2 (OMIM #611762) is a rare autosomal dominant autoinflammatory disorder caused by mutations in the *NLRP12* gene [24,25], with most reported variants clustered in exon 3. NLRP12, a member of the NOD-like receptor (NLR) family, functions primarily as a negative regulator of inflammation by suppressing the NF-κB pathway and reducing the production of proinflammatory cytokines such as IL-1β, IL-6, and IL-8 [26,27].

Clinically, patients develop recurrent urticarial eruptions, fever, arthralgia or arthritis, myalgia, and lymphadenopathy following exposure to cold or mild cooling. In approximately 66% of cases, rashes occur within several hours after cold exposure, accompanied by elevated CRP and ESR. The age of onset ranges from infancy to middle age, with a slight male predominance; about half have a positive family history, and some experience abdominal or chest pain, headache, or sensorineural hearing loss [24,26,28,29].

Functional studies indicate that NLRP12 interacts closely with the NLRP3 inflammasome. Although its precise role remains debated, NLRP12 can either assemble an inflammasome complex or modulate its activity. It promotes caspase-1 activation and IL-1β maturation, accompanied by redox alterations [30,31,32]. Caspase-1 also induces apoptosis and weakens the negative regulation of TNF-induced NF-κB signaling [7,33,34]. *NLRP12*-deficient mice exhibit attenuated systemic inflammation and neutrophil infiltration [35].

There is no standardized treatment for FCAS2. Glucocorticoids, antihistamines, and NSAIDs may provide temporary relief but rarely prevent recurrence. Owing to the central role of IL-1β, IL-1 antagonists such as anakinra and canakinumab have shown marked clinical efficacy in several cases, whereas TNF-α inhibitors yield inconsistent results [7,24,36,37].

### 3.3. NLRC4-FCAS4

FCAS associated with *NLRC4* mutations is exceedingly rare worldwide. The first kindred was reported in Japan in 2014, showing infant-onset cold-induced fever, urticarial rash, and arthralgia [38]. A similar phenotype was later identified in a Dutch family, with some patients presenting conjunctivitis, arthritis, or colitis [39]. Sporadic cases with unexplained sensorineural hearing loss have also been described [8].

The NLRC4 protein is a key innate immune sensor that assembles its inflammasome upon detecting bacterial components [40]. Pathogenic gain-of-function variants can trigger inflammasome activation independently of infection, leading to excessive secretion of IL-1β and IL-18 [41,42]. Clinical severity varies widely, ranging from mild recurrent rash and arthralgia (FCAS4) to macrophage activation syndrome (MAS) or autoinflammation with infantile enterocolitis (AIFEC) [43].

Functional studies demonstrate that mutant *NLRC4* enhances inflammasome activity, increasing IL-1β, IL-6, and caspase-1 activation [43]. Under cold conditions, inflammasome overactivation is associated with HSC70 disinhibition [44]. Elevated IL-18 levels may serve as a biomarker [39,41]. Skin biopsies reveal lympho-histiocytic infiltrates, distinct from the neutrophilic lesions typical of FCAS1 [45]. In vitro models further show that mutant NLRC4 upregulates IL-1β, which promotes neutrophil-derived IL-17A production, amplifying inflammation [38].

Most FCAS4 patients respond well to NSAIDs; severe cases may require corticosteroids, colchicine, or IL-1 inhibitors, whereas TNF-α antagonists are rarely used [8,43].

### 3.4. PLCG2-FCAS3

Pathogenic dominant mutations in the phospholipase Cγ2 (*PLCG2*) gene are linked to a wide range of immune dysregulation, extending from allergic and immunodeficient phenotypes to autoimmune and autoinflammatory manifestations. These variants underlie two related conditions: PLCG2-associated antibody deficiency and immune dysregulation (PLAID, OMIM 614468) and its autoinflammatory counterpart APLAID (OMIM 614878). PLAID, also termed FCAS3, is a rare autoinflammatory disorder characterized by cold-induced urticaria, humoral immunodeficiency, cutaneous granulomas, and autoimmune manifestations [46,47,48].

In 2012, Ombrello et al. [9] first described PLAID patients presenting early-onset cold-induced rash, decreased serum IgM and IgA, reduced class-switched memory B cells, and diminished circulating NK cells. Clinically, PLAID differs from typical ColdU, showing negative CSTT results and symptoms triggered mainly by cold air rather than direct contact with cold objects. Mutations cluster within in-frame deletions of the autoinhibitory cSH2 domain of *PLCG2*, disrupting its regulatory function, and leading to constitutive enzymatic activation at sub-physiological temperatures [31,49,50]. In vitro studies revealed that mutant PLCγ2 exhibits enhanced basal and receptor-dependent activity, whereas patient immune cells show attenuated receptor responses, suggesting desensitization due to chronic low-level activation [9].

B and NK cells display abnormal calcium flux: B cells are hyporesponsive at ambient temperature but become spontaneously activated with elevated intracellular Ca^2+^ at ~20 °C. Expression of mutant *PLCG2* in mast cells induces spontaneous degranulation at sub-physiologic temperatures, unlike wild-type cells, indicating temperature-dependent signaling imbalance. This mechanism causes B-cell hyperactivation, reduced antibody production, impaired immune tolerance, and aberrant mast-cell degranulation, manifesting as cold-induced urticarial lesions [9]. In some cases, PLCG2 variants produced greater effects in natural killer cells than in B cells [51].

Thus, PLAID represents a distinct subtype that bridges dysregulated adaptive immune signaling with aberrant mast-cell activation, characterized by temperature-sensitive receptor signaling imbalance. This finding expands the immunological spectrum of ColdU, suggesting that the molecular basis of cold-induced reactions may extend beyond inflammasome and IgE-mediated pathways to broader B-cell signaling abnormalities.

Recent studies further indicate that not all PLAID cases are attributable to *PLCG2* mutations; some lack abnormal transcripts, implying potential non-genetic mechanisms (e.g., novel autoantibodies), locus heterogeneity, or somatic mutations within hematopoietic or mast-cell lineages that generate cold-sensitive cellular subsets [52].

## 4. FXII-Associated Cold Autoinflammatory Syndrome (FACAS)

FXII plays multiple roles in coagulation, fibrinolysis, complement activation, and the kallikrein–kinin system. Mutations in the *F12* gene are linked to hereditary angioedema with normal C1 inhibitor (FXII-HAE) [53]. In 2020, FXII-associated cold autoinflammatory syndrome (FACAS) was first described as an autosomal dominant disorder caused by a heterozygous *F12 p.W268R (T859A)* mutation. Affected members across four generations developed generalized urticarial eruptions, chills, arthralgia, and low-grade fever following cold exposure [10].

Functional studies demonstrated that the mutation enhances FXII autocatalytic cleavage and spontaneous activation, leading to sustained contact system activation characterized by decreased prekallikrein, increased cleavage of high-molecular-weight kininogen (HMWK), and elevated bradykinin (BK). Neutrophils serve as a local source of FXII, while IL-1β is upregulated in lesional tissue and monocytes exposed to mutant FXII, suggesting that FXII activation extends beyond vascular pathways to direct cytokine-driven inflammation. BK stimulates monocytes via the B2 receptor to release IL-1β, which in turn promotes FXII expression and activation, forming an amplifying loop of vascular permeability and inflammation [10,54,55,56]. Additionally, BK induces mast-cell degranulation with heparin and polyphosphate release, further enhancing FXII activation in a local positive feedback cycle. Mast-cell enrichment and IL-1β–mediated activation jointly drive urticarial lesions in FACAS [57,58,59].

Histopathology shows dermal edema with perivascular macrophage and neutrophil infiltration. Lesions typically appear 10–30 min after cold exposure, last for several hours, and are CSTT-negative. FACAS responds poorly to second-generation antihistamines but shows marked improvement with bradykinin B2 receptor antagonists (icatibant) or IL-1 inhibitors [10]. Although it shares IL-1β–mediated inflammation with CAPS, its underlying mechanism differs and overlaps with the kallikrein–kinin cascade seen in HAE.

## 5. Discussion

As the molecular mechanisms of FCU are increasingly elucidated, diagnostic strategies are shifting from symptom-based classification toward genetic and immunologic confirmation. The CSTT remains a key initial screening tool: patients who are CSTT-positive and respond well to antihistamines are typically diagnosed with ACU or FP-ACU, whereas CSTT-negative patients with fever, arthralgia, or elevated inflammatory markers should be evaluated for inflammasome-related subtypes. Functional assays assessing NLRP3 inflammasome activation have been shown to aid diagnosis even in patients without identifiable mutations [60]. In addition, high-depth exome or whole-genome sequencing can detect low-level somatic mosaicism, which may be crucial for diagnosing mild or late-onset FCAS cases [14,22,23].

A considerable subset of ColdU patients presents with CSTT-negative results. Recently, Ahsan et al. proposed the concept of ‘Atypical subtypes of ColdU’ and suggested subclassification based on clinical triggers and systemic manifestations. The modified cold stimulation test (mCSTT) improves diagnostic sensitivity, while the inclusion of family history and genetic testing enhances diagnostic standardization [61].

The pronounced clinical heterogeneity of FCU poses additional diagnostic challenges. All FCAS subtypes feature cold-induced rash and systemic inflammation, but gastrointestinal symptoms are more frequent in NLRP12-AID, whereas headache and dizziness appear less common than in NLRP3-AID [62]. FACAS should be differentiated from HAE, while PLAID requires distinction from immunodeficiency or chronic spontaneous urticaria. Establishing a diagnostic framework centered on inflammatory mechanisms can assist in recognizing “atypical ColdU” and reduce the likelihood of misdiagnosis.

From a therapeutic perspective, H1 receptor antagonists remain the cornerstone of prophylactic treatment for FACU and FP-ACU. Patients at risk of systemic cold exposure should have access to epinephrine autoinjectors [63]. Cold exposure may induce novel auto-allergens recognized by IgE bound to mast cells, triggering degranulation. The efficacy of omalizumab in approximately half of antihistamine-refractory ColdU patients supports the role of IgE in disease pathogenesis [64,65,66]. Anti-KIT antibody, barzolvolimab has shown potential in antihistamine-resistant ACU, suggesting therapeutic promise for mast-cell-mediated cold-induced diseases, although larger studies are needed [67].

IL-1β antagonists remain the principal targeted therapy for FCAS. In some cases, IL-6 levels rise significantly after cold exposure, and IL-6 inhibition may be effective when IL-1 blockade fails [68,69,70]. Although numerous NLRP3 inhibitors are under development, no clinical trials have yet assessed their use in FCAS; preventive application may offer new management opportunities [71,72]. Currently, NSAIDs with or without corticosteroids, remain standard for symptom control, while biologics—including IL-18–targeted therapy—are reserved for severe cases [7,73,74].

Management of PLAID requires addressing both immunodeficiency and cold avoidance [75]. Glycopyrrolate or high-dose corticosteroids may be considered when H1 blockers are ineffective [47,76,77]. FACAS patients can benefit from combined therapy with bradykinin B2 receptor antagonists and IL-1 inhibitors, while plasma kallikrein inhibitors and FXIIa inhibitors represent promising therapeutic candidates [78,79].

## 6. Conclusions

Over the past two decades, the diagnostic spectrum of FCU has expanded substantially. Differences in pathogenesis and therapeutic responsiveness among subtypes underscore the importance of integrating detailed clinical assessment, laboratory investigation, and molecular analysis. Although several causative genes have been identified, a considerable proportion of patients still lack a definitive molecular diagnosis, suggesting that additional pathogenic pathways remain to be uncovered.

Future studies should focus on the interplay between cold stimulation and immune activation. In particular, understanding how cold-sensing ion channels regulate immune cell responses and interact with innate immune receptor signaling will provide important mechanistic insights. Elucidating the crosstalk between mast cells and inflammasomes in cold-induced reactions—and their differential roles across FCU subtypes—will refine disease stratification. Integration of multi-omics approaches with artificial-intelligence-based analysis is expected to uncover molecular subgroups of cold-responsive diseases, thereby facilitating precision diagnosis and personalized therapy for cold urticaria.

## Figures and Tables

**Table 1:** Classification and management of familial cold urticarias.

Characteristic	FACU	FP-ACU	FCAS1/ NLRP3	FCAS2/ NLRP12	FCAS4/ NLRC4	PLAID/ FCAS3	FACAS
**Pathway involved**	Mast Cell Activation	Mast Cell Activation	NLRP3 Inflammasome	NLRP12 Inflammasome	NLRC4 Inflammasome	B-cell signaling and functional abnormalities	Kallikrein–kinin system
**Inheritance**	AD	Familial tendency	AD	AD	AD	AD	AD
**Gene**	Unknown	Unknown	NLRP3	NLRP12	NLRC4	PLCG2	F12
**Protein involved**	-	-	Cryopyrin	NLRP12	NLRC4	PLCγ2	Factor XIl
**CSTT**	Negative	Positive	Negative	Negative	Negative	Negative	Negative
**Onset of rash after cold exposure**	0 min to 30 min	0 min to 30 min	1–2 h	1–2 h	<5 min	<5 min	10–30 min
**Duration of episodes (range)**	Minutes to several hours	Minutes to several hours	30 min to 72 h	Hours to days	Hours to days	30–60 min; resolution < 1 h after rewarming	1 to several hours after rewarming
**Skin manifestations**	Pruritus, erythema, and urticaria	Itchy wheals and/or angioedema	Tender, nonpruritic, urticaria-like rash, erythematous papules/plaque	Urticaria-like rash, erythematouspapules/plaques	Urticaria-like rash, erythematouspapules/plaques	Urticarial, pruritic, erythema, with or without angioedema, burning	Generalized wheals/urticarial rash, nonpruritic, burning sensation, no angioedema
**Histopathology**	Mast cell infiltrate with degranulation post-cold challenge	Mast cell infiltrate with degranulation post-cold challenge	Dermal edema and perivascular infiltrates of primarily neutrophils often observed surrounding sweat glands.	Neutrophil infiltrates	Predominantly lymphocytic and histiocytic infiltration	Mast cell infiltrate with degranulation post-cold challenge	Dermal edema with sparse perivascular macrophage/neutrophil infiltrates; IL-1β expression
**Other symptoms**	Angioedema (Oropharyngeal swelling and/or abdominal pain) and syncope	Atopy, anaphylaxis	Fever/chills, arthralgia, lethargy, headache, conjunctivitis, and fatigue.	High fever (over 40 °C), chest pain, abdominal pain, conjunctivitis, arthralgia, loss of strength, splenomegaly, and hepatomegaly	Recurrent fever/chills, arthralgia, myalgia, headache, fatigue	Atopy, granulomatous rash, autoimmune thyroiditis, the presence of antinuclear antibodies, sinopulmonary infections, and common variable immunodeficiency	Arthralgias, chills, headache, fatigue, and subfebrile temperatures in the evening
**Usual age of onset (years)**	Childhood	Childhood	Infancy	Variable	Infancy	Infancy	Variable
**Biomarkers**	-	IgE ↑ Variable	IL-1β ↑, IL-6 ↑, SAA ↑, neutrophilia ↑, Serum inflammatory markers ↑	IL-1β ↑, Serum inflammatory markers ↑	IL-1β ↑, IL-18 ↑	IgM ↑, IgA ↓, Nearly all patients had marked diminution of class-switched B-cells	Plasma prekallikrein ↓, BK↑, IL-1β ↑, SAA ↑, S100 A12/ ↑, S100 A8/9 ↑
**Treatment response**	H1 antihistamines, omalizumab	H1 antihistamines, omalizumab	Anakinra, rilonacept, canakinumab	Nonsteroidal anti-inflammatory drugs, anakinra, rilonacept, canakinumab	Nonsteroidal anti-inflammatory drugs, anakinra, canakinumab	Supportive; H1-receptor antagonists, immunoglobulin replacement, glycopyrrolate or high-dose corticosteroids	Icatibant, anakinra

AD, autosomal dominant; FACU, Familial Atypical Cold Urticaria; FP-ACU, Familial Predisposed Acquired Cold Urticaria; FCAS1/NLRP3, Familial Cold Autoinflammatory Syndrome type 1 (NLRP3-associated); FCAS2/NLRP12, Familial Cold Autoinflammatory Syndrome type 2 (NLRP12-associated); FCAS4/NLRC4, Familial Cold Autoinflammatory Syndrome type 4 (NLRC4-associated); PLAID/FCAS3, PLCG2-Associated Antibody Deficiency and Immune Dysregulation; FACAS, FXII-Associated Cold Autoinflammatory Syndrome; CSTT, Cold Stimulation Test; ↑: Increased; ↓: Decreased.

## Data Availability

No new data were created or analyzed in this study.
